# How the pilidium larva grows

**DOI:** 10.1186/2041-9139-5-13

**Published:** 2014-04-01

**Authors:** April M Bird, George von Dassow, Svetlana A Maslakova

**Affiliations:** 1 Oregon Institute of Marine Biology, PO Box 5389, Charleston, OR 97420, USA

**Keywords:** Larval growth, Stem cells, Ciliated epithelia

## Abstract

**Background:**

For animal cells, ciliation and mitosis appear to be mutually exclusive. While uniciliated cells can resorb their cilium to undergo mitosis, multiciliated cells apparently can never divide again. Nevertheless, many multiciliated epithelia in animals must grow or undergo renewal. The larval epidermis in a number of marine invertebrate larvae, such as those of annelids, mollusks and nemerteans, consists wholly or in part of multiciliated epithelial cells, generally organized into a swimming and feeding apparatus. Many of these larvae must grow substantially to reach metamorphosis. Do individual epithelial cells simply expand to accommodate an increase in body size, or are there dividing cells amongst them? If some cells divide, where are they located?

**Results:**

We show that the nemertean pilidium larva, which is almost entirely composed of multiciliated cells, retains pockets of proliferative cells in certain regions of the body. Most of these are found near the larval ciliated band in the recesses between the larval lobes and lappets, which we refer to as axils. Cells in the axils contribute both to the growing larval body and to the imaginal discs that form the juvenile worm inside the pilidium.

**Conclusions:**

Our findings not only explain how the almost-entirely multiciliated pilidium can grow, but also demonstrate direct coupling of larval and juvenile growth in a maximally-indirect life history.

## Background

It is said that no animal cell divides while ciliated [1,2]. Although many protists retain motile cilia as they divide, in proliferative animal cells the single motile or non-motile cilium must be resorbed during mitosis, then regenerated [3-5]. The sole exceptions we know of are the spermatocytes of some insects, for example, [6]. Meanwhile, multiciliated cells in animals are terminally differentiated, and, as such, do not divide [7]. How then do multiciliated animal epithelia grow and repair damage? Postembryonically, animal tissues must accomplish differentiation and functionality while retaining the capacity for cell proliferation. An obvious example is the vertebrate airway epithelium, which is lined by cells bearing multiple cilia, but must retain the ability to replace damaged cells [8,9]. Another example is the planktonic larvae of benthic marine invertebrates, many of which swim and feed using ciliated organs, and, at the same time, dramatically increase in size over the course of larval life [10]. Larval epithelia of deuterostomes, such as sea urchins, are typically composed of uniciliated cells that can divide after temporarily resorbing the cilium [5]. Such larvae require very many cells to swim and feed efficiently. Many lophotrochozoan larvae, on the other hand, swim and feed even at comparatively small cell number by using multiciliated cells [10]. Larvae of annelids and mollusks that swim using multiciliated cells typically also have non-ciliated regions, which could be responsible for the growth of the ciliated organs [10-12].

The nemertean pilidium larva, on the other hand, appears to be composed entirely of multiciliated cells with the exception of specialized sensory cells in the apical organ and the ciliated band [13]. Yet the pilidial surface area increases by a factor of ten to one hundred during its larval life [14]. How then does the pilidium larva grow? There are three possibilities. First, it could be that the pilidium’s larval body is essentially eutelic, like many small animals: perhaps growth or shape change of individual cells, as opposed to cell division, accounts for the increase in surface area of the larva. This would seem to preclude repair, and, although plants manage such feats through vacuolar expansion, seems unlikely to account for all of the growth needed to expand the surface of a typical nemertean blastula to its eventual area. Second, perhaps nemerteans possess some mechanism to bypass ciliation constraints, such that multiciliated cells in the pilidial epithelium are capable of transdifferentiation and cell division. This possibility seems remote, although, if it is indeed simply the multiplicity of basal bodies that is the root of the inferred constraint, surely some animal must have evolved a solution, such as to degrade the extras (as the wasp *Muscidifurax* does during early mitoses [15]) or start afresh (as rodents do post-zygotically [16]).

The third possibility is that there could be some population of non-ciliated or uniciliated cells among or underneath the multiciliated cells in the pilidium that, meristem-like, remain able to divide and contribute to the growing larval body. This is what we show here. Using complementary labeling methods and scanning electron microscopy, we show that the pilidium’s equivalent of meristems are located at the clefts between the lobes that bear the ciliary band; we call these sites ‘axils’. The axillary growth zones contribute to both larval structures and juvenile rudiments. Indeed, aside from the stomach and a few other organs, it appears that most of the juvenile descends from these few cells. But perhaps it is more remarkable that the majority of this growing, feeding larval body is also produced by cells set aside during early development for indefinite proliferation.

## Methods

### Larval culture

Adults of *Micrura alaskensis* were collected using a shovel from intertidal sand flats in Charleston, OR, USA. Gravid males and females were minced to release gametes; and oocytes inseminated with dilute suspension of sperm. Larvae were reared in glass containers of filtered natural sea water (FSW) kept at ambient sea temperature, with constant stirring, regular water changes, and feeding on *Rhodomonas lens* (CCMP739), as described by Maslakova [14].

### Microinjections of fluorescent markers

Zygotes of *M. alaskensis* were de-jellied by repeatedly passing them through a hand-pulled mouth pipet cut to slightly larger than the egg diameter. To prevent de-jellied eggs from sticking to plastic, Petri-dishes were coated with 1% BSA in FSW before adding eggs. For injection, de-jellied eggs were arranged in an uncoated coverslip-bottom dish that had been cleaned with 95% ethanol. Ethanol-cleaned glass is sticky enough to enable microinjection but allows release of injected embryos with gentle swirling. Eggs were injected either with polyadenylated mRNA encoding EMTB-3xGFP (Ensconsin microtubule binding domain), as described by von Dassow *et al*. [17], at a needle concentration of 50 to 100 ng/μl in RNase-free water, or with FITC-dextran at 5 μg/μl in sterile aspartate injection buffer (100 mM potassium aspartate, 50 mM KCl, 10 mM HEPES pH 7.4). Eggs were injected with 1 to 2% of the cell volume using needles made from 1 mm OD filament-containing capillaries on a micropipette puller (P-94; Sutter Instruments Co., Novato, CA, USA). Injections were performed on an inverted microscope (Leica DMIL; Leica Microsystems, Buffalo Grove, IL, USA) using a hanging-joystick oil hydraulic micromanipulator (Narishige, East Meadow, NY, USA) and pressure microinjector (PMI-200; Dagan Corp., Minneapolis, MN, USA). Labeled embryos were cultured in 35 or 60 mm Petri-dishes at ambient sea temperature with regular water changes and feeding on *Rhodomonas lens* for up to six weeks and imaged live.

### BrdU labeling

For BrdU pulse experiments four-day old pilidia of *M. alaskensis* were incubated with 0.1 mg/ml BrdU (Sigma, St. Louis, MO, USA; B5002) in FSW for 24 hours. After that, larvae were relaxed in 0.37 M MgCl_2_ and fixed in 4% paraformaldehyde (Electron Microscopy Science, Hatfield, PA, USA) in FSW for 30 to 45 minutes. For BrdU pulse-chase experiments, six-day old larvae were labeled with BrdU for six hours, and subsequently cultured in one-gallon glass containers, as described above, for three days (short chase) and fourteen days (long chase), and then fixed. Preserved larvae were washed in several changes of PBS (pH 7.4, Fisher Scientific, Waltham, MA, USA), incubated in approximately 1.0 N HCl for 15 to 25 minutes to denature DNA, and washed in several changes of 0.1 M Na_2_B_4_O_7_ (over 20 minutes) to neutralize the acid. Larvae were permeabilized in several changes of 0.1% Triton X-100 in PBS (PBT) over 30 minutes, incubated with 5 to 10% normal goat serum (in PBT with 0.1% BSA) for two hours at room temperature to block non-specific binding. Larvae were incubated with a mouse anti-BrdU monoclonal antibody (Becton Dickinson, Franklin Lakes, NJ, USA) diluted 1:100 in PBT/BSA overnight at 4°C, washed in several changes of PBT/BSA and incubated with Alexa Fluor 488 goat-anti-mouse antibody diluted 1:500 in PBT for two hours at room temperature. To visualize all nuclei, larvae were additionally labeled with Hoechst 33342 (500 nM) in PBT at room temperature for 30 to 40 minutes. Labeled larvae were washed several times in PBS, and mounted on cover glass coated with poly-L-lysine in Vectashield (Vector Laboratories, Burlingame, CA, USA) or 75% glycerol in PBS. Slide preps were sealed with nail polish (Wet N Wild, Los Angeles, CA, USA) and imaged on a confocal microscope.

### Confocal microscopy

Pilidia labeled with EMTB-3xGFP and FITC-dextran were imaged live. To slow down ciliary motion, larvae were gently trapped on slides under a coverglass supported by vacuum grease, and stunned by addition of 0.1% NaN_3_. Larvae were imaged using an Olympus FluoView 1000 laser scanning confocal system (Olympus America, Center Valley, PA, USA) on an inverted microscope (Olympus IX81) using Apo 40× water (NA 1.15) or Plan Apo 60× water (NA 1.2) objectives. Fixed larvae were imaged with UPlanFL 40× oil (NA 1.3) or Plan Apo 60× oil (NA 1.42) objectives. Confocal stacks were imported into ImageJ for processing, and false-coloring was applied in PhotoShop CS6.

### Scanning electron microscopy

Two- to four-week-old pilidia of *M. alaskensis* were relaxed for 5 to 10 minutes in a 1:1 mixture of FSW and 0.34 M MgCl_2_, then killed by adding a drop of 1% formalin, and fixed by first replacing this mixture with a volume of 2.5% glutaraldehyde in 0.2 M Millonig’s phosphate buffer pH 7.4 or 0.2 M sodium cacodylate buffer pH 7.4 (Electron Microscopy Sciences, Hatfield, PA, USA), and after a few minutes, adding an equal volume of 4% OsO_4_ (Electron Microscopy Sciences, Hatfield, PA, USA) for a final concentration of 1.25% glutaraldehyde and 2% OsO_4_. Larvae fixed for one to two hours were rinsed in several changes of deionized water, dehydrated through an ethanol series (30% to 50% to 70%), and stored in 70% ethanol until further processing. Larvae were further dehydrated to 100% ethanol (through a series with 10% concentration increment), dried using a CO_2_ EMS K850 Critical Point Drier, then sputter-coated with gold using an Emscope SC500, and examined using a Tescan Vega II scanning electron microscope.

## Results and discussion

### Growth of the nemertean pilidium larva is largely due to cell division in the axils

**Figure 1:**
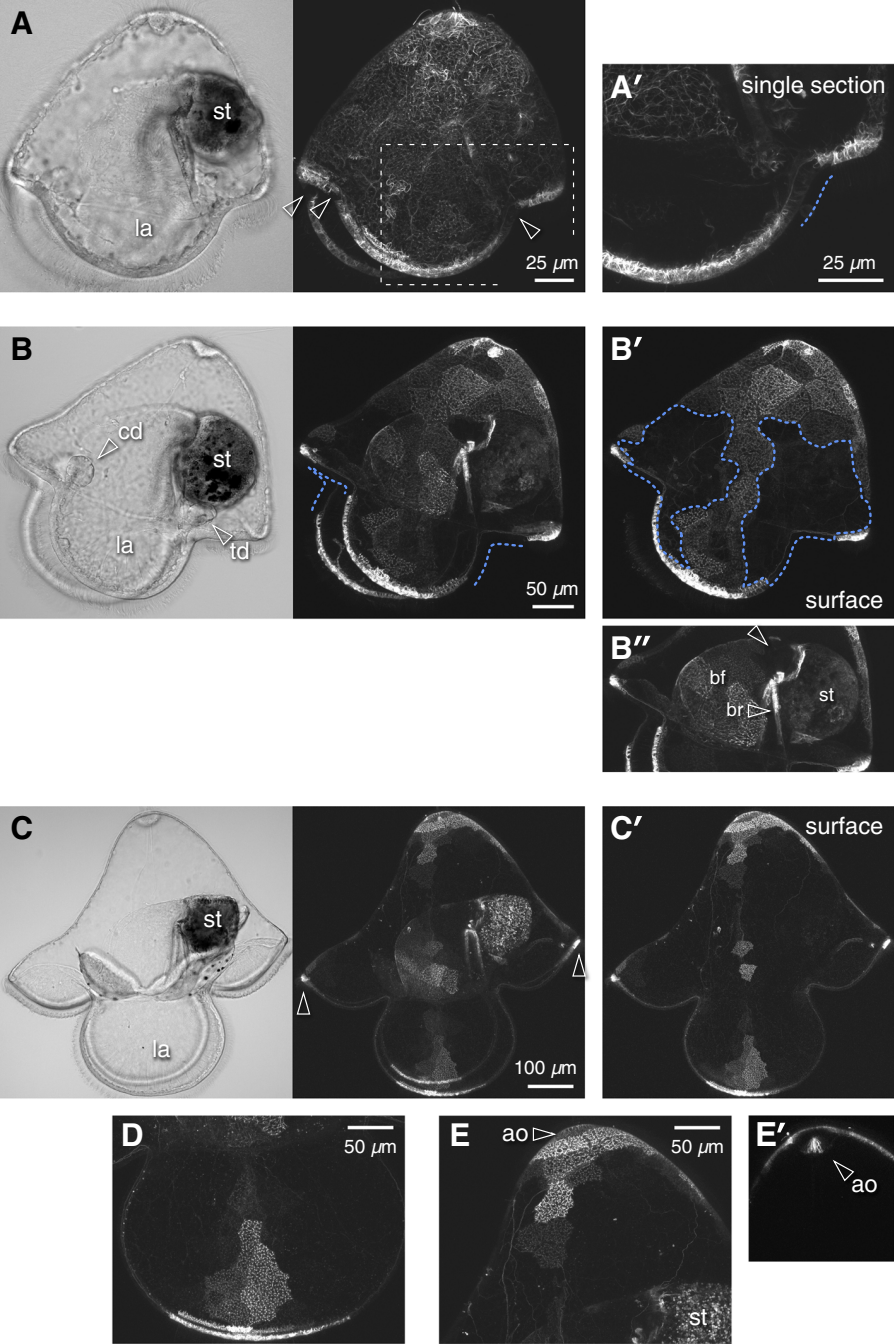
**Persistence of embryonically-expressed GFP in pilidia of *M. alaskensis.*** All panels show larvae raised from zygotes injected with EMTB-3xGFP mRNA, anterior left and apical up. Note that in small-volume unstirred cultures, developmental rate is slower compared to times reported by Maslakova [14]. Left panels of **(A)** to **(C)** show transmitted light image, right panels show GFP fluorescence. Regions of reduced GFP fluorescence are attributed to cell division. **(A)** Young pilidium, 11 days old, imaginal discs not yet invaginated. Small dark patches have appeared in the axils (arrowheads) anterior and posterior to the lateral lappets (la). st = stomach. **(*A’*)** Single confocal section through posterior axil (corresponds to dashed outline in **(A)**; extent of the dark patch indicated by dashed line. **(B)** 21-day-old larva with cephalic and trunk discs (cd, td); dashed lines indicate the extent of the dark axillary regions within the larval primary ciliated band. Large dark patches (outlined in **(*B’*)**; near-surface projection) include the axils and nearly half of the episphere. **(*B”*)** Projection of the sections through the buccal cavity; arrowhead indicates dark zone near stomach entrance. bf = buccal funnel, br = buccal ridge. **(C)** 34-day-old torus-stage pilidium. Only small patches of the lappets (la) and the apex, the anterior and posterior lobes (arrowheads), and about half the buccal funnel, retain label; **(*C’*)** shows near-surface projection. **(D)** Magnification of near-side lappet from C. **(E)** Magnification of apex and apical organ (ao) from **(C)**. Innermost whorls of cells in the apical organ retain label, as seen on a medial slab through the apical organ **(*E’*)**, while peripheral cells of the apical organ are dark.

**Figure 2:**
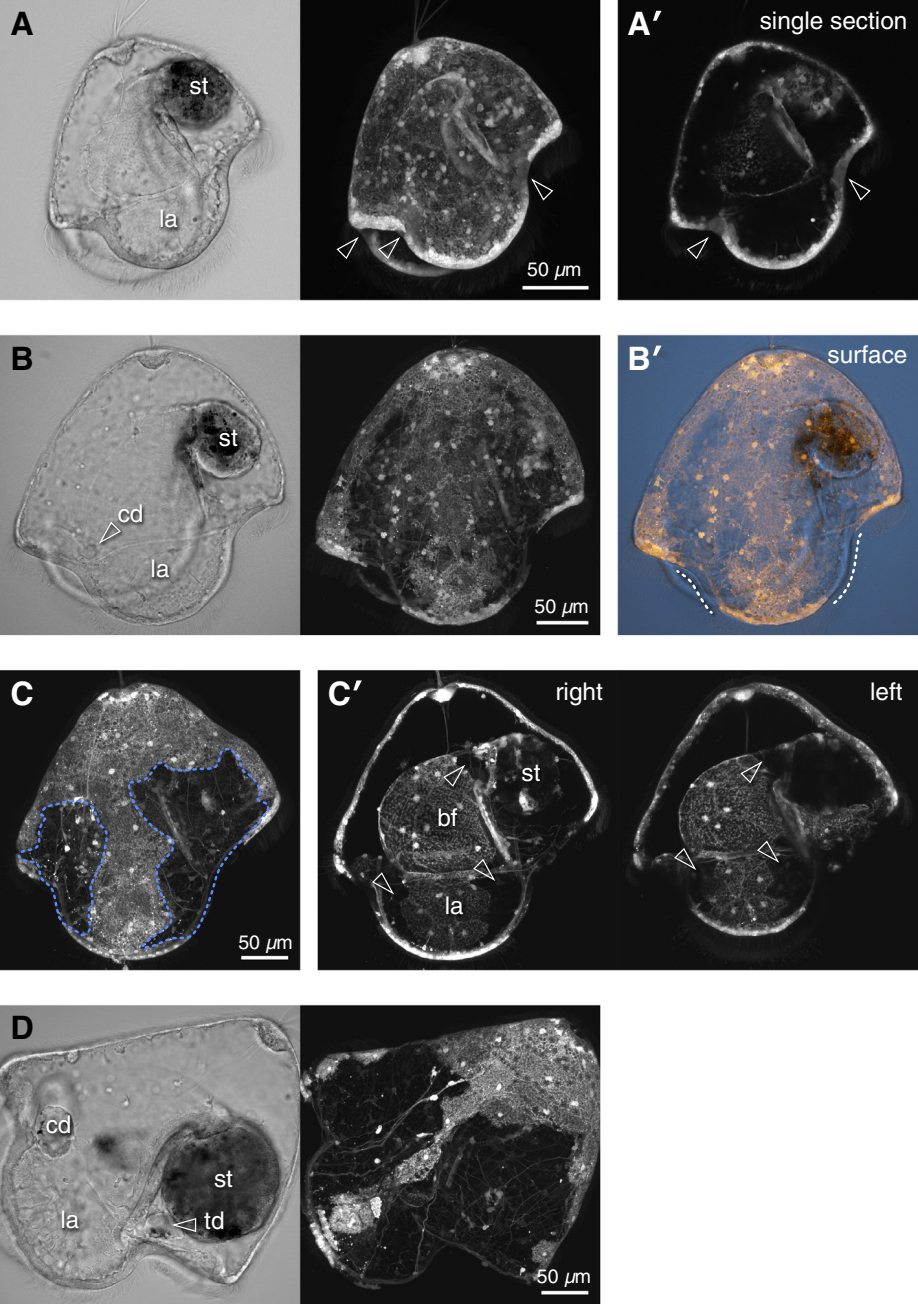
**Persistence of FITC-dextran in pilidia of *M. alaskensis.*** All panels show larvae derived from zygotes injected with FITC-dextran; anterior left, apical up; cd = cephalic disc, la = lappet, st = stomach, td = trunk disc, bf = buccal funnel, br = buccal ridge. **(A)** Feeding pilidium, eight days old, before imaginal disc formation. Small dark zones developing in anterior and posterior axils (arrowheads); **(*A’*)** Single section at a level which includes entire near-half ciliary band. **(B)** Pilidium with developing cephalic discs, 16 days old. Dark zones have spread from axils to cover nearly half the episphere; **(*B’*)** shows the near surface only, and dashed lines indicate the unlabeled region of the ciliary band. **(C)** Another 16-day-old larva from the same cohort, in which both cephalic and trunk discs had formed (not visible); dashed outlines indicate the dark zones, that is growing regions of larval epidermis. **(*C’*)** Projection of subsets covering the right and left halves of the buccal funnel, showing dark patches (arrowheads) both on the inner lappet surface, anterior and posterior, and around the stomach entrance. **(D)** 26-day-old pilidium, with two pairs of discs; more than half of the larval epidermis consists of dark (that is, post-embryonically proliferated) cells.

Our first indication that cell proliferation, rather than changes in cell shape, is responsible for larval growth in the pilidium came from experiments in which we injected zygotes of the heteronemertean *Micrura alaskensis* with mRNA encoding a fluorescent protein fusion to the microtubule binding domain of Ensconsin (EMTB-3xGFP). Surprisingly, this label remained bright for many weeks, even months. However, it appeared to be progressively diluted in some areas of the larval body (Figure 1). Dilution, not differential turnover, leads to the observed pattern, because we observed identical patterns with inert tracers like FITC-dextran (Figure 2) or with several other long-lived fluorescent proteins. We interpret dilution of the label as evidence of cell proliferation with growth.

Early in larval development the areas of label dilution (dark regions) were detectable in the recesses between the pilidial lobes and lappets in vicinity of the main larval ciliated band (Figures 1A and 2A, arrowheads). We named these regions ‘axils’ (Latin for ‘pit’), drawing an analogy to plant leaf axils which harbor pluripotent cells of axillary meristems. The pilidium is bilaterally symmetrical. Accordingly, there are two anterior axils: one on each side of the larva at the inflection points between the anterior lobe and the lateral lappets, and two posterior axils at the points of inflection between the posterior lobe and the lateral lappets. The dark areas got progressively larger as the pilidia grew (Figures 1B and 2C, marked with dashed line). By the torus stage (Figure 1C; staging scheme follows Maslakova [14]), the larva is nearly four times bigger in linear dimensions compared to the young pilidium (compare to Figure 1A), thus the surface area increase is on the order of 16. Remarkably, at this point the remaining brightly labeled areas of the larval epidermis were about the same in absolute size (for example, compare the length of the brightly labeled area of the ciliated band along the lateral lappet on Figure 1B and D). This means that the increase in surface area was largely accounted for by the dark regions, which, indeed, occupied the majority of the larval epidermis at advanced developmental stages (Figures 1C, D, and 2D). In addition to the axils, dilution of fluorescent label was noticeable near the entrance to the pilidial stomach in the wall of the buccal cavity (Figure 1B”), and at the periphery of the apical organ (Figure 1E). This effect was not label-specific, because injection of FITC-dextran into zygotes of *M. alaskensis* revealed a similar pattern (Figure 2).

**Figure 3:**
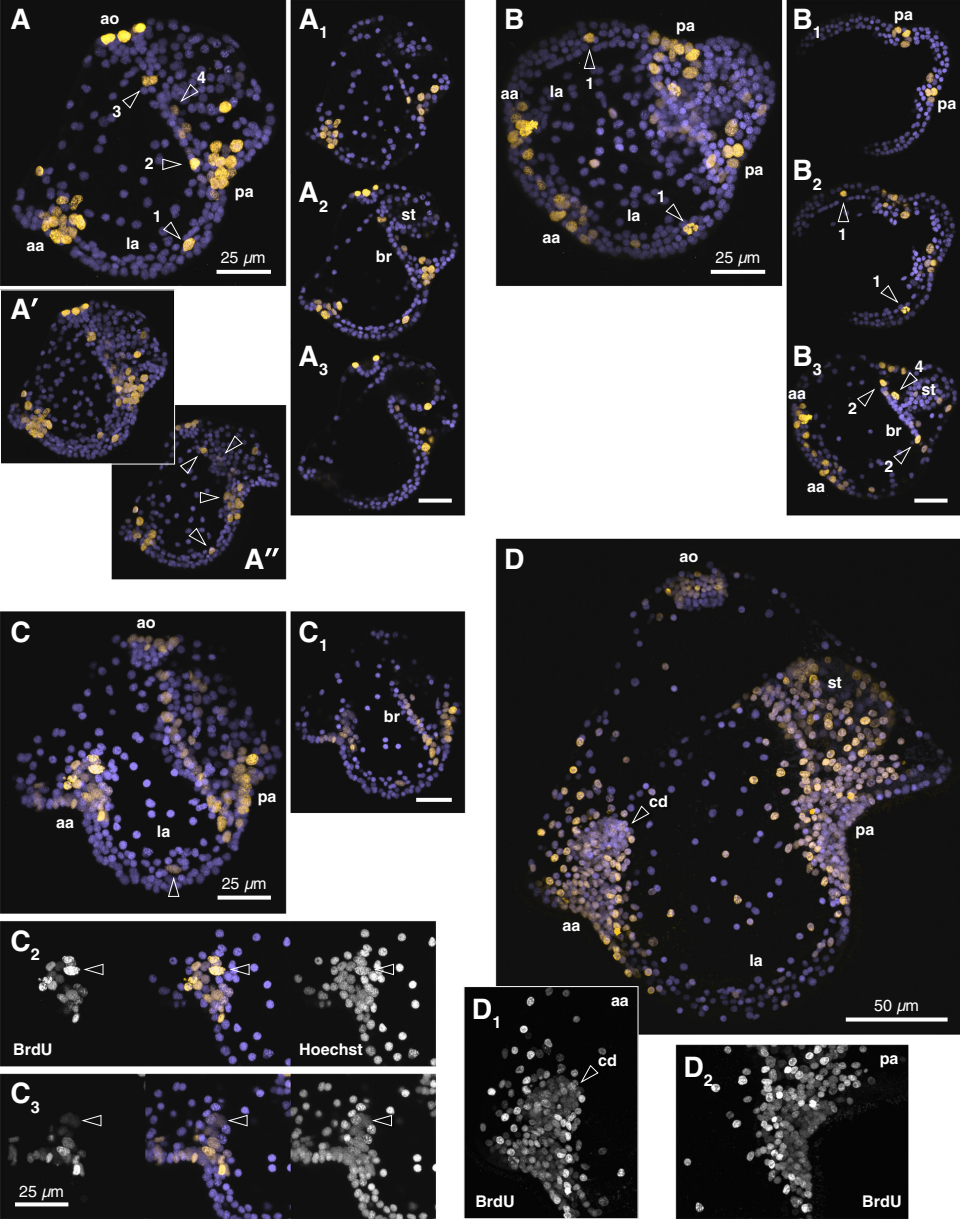
**Sites of BrdU incorporation in the pilidium.** All: anterior left, apical up; purple = Hoechst, yellow = BrdU, aa = anterior axil, pa = posterior axil, ao = apical organ, la = lappet, st = stomach, br = buccal ridge. **(A, B)** Young pilidia (four days old) incubated for 24 hours in BrdU, then fixed immediately, lateral **(A)** and oral **(B)** views. **(A)** shows the nearer-to-lens half of the larva; **(*A’*)** projection of entire larva; **(*A”*)** other half of the larva, to illustrate bilateral symmetry of labeled populations. **(*A***_***1 ***_**to *A***_***3***_**, *B***_***1 ***_**to *B***_***3***_**)**: three to five section subsets emphasizing specific features. The most prominent labeling is in anterior and posterior axils and apical organ; other specific sites include a mesenchymal cell beneath the lappet ciliary band (arrowhead 1), a single cell at the end of the buccal ridge (arrowhead 2), a single cell on either side of the stomach entrance (arrowhead 3), and two to three mesenchymal cells behind the buccal ridges (arrowhead 4). **(C)** 9-day-old pilidium with nascent cephalic imaginal discs, labeled with six-hour BrdU treatment on day 6. **(*C***_***1***_**)** Projection of seven 1-μm sections including the buccal ridge; note the row of BrdU-labeled cells alongside unlabeled nuclei. **(*C***_***2***_**, *C***_***3***_**)** grayscale and false-colored substacks (11 1-μm sections) of anterior axil **(*C***_***2***_**)** and cephalic disc beneath **(*C***_***3***_**)**. Some cells retain bright labeling (arrowhead in ***C***_***2***_); others, especially in the disc, have diluted the BrdU substantially (arrowhead in ***C***_***3***_). **(D)** 20-day-old pilidium labeled with six-hour BrdU pulse on day 6; projection through half the larva nearer to the lens. Axillary BrdU labeling is shown on grayscale insets **(*D***_***1***_**)** (anterior) and **(*D***_***2***_**)** (posterior). A constellation of few bright epithelial cells surrounds the much-diluted cluster of axillary cells and their progeny, including the cephalic imaginal disc (cd).

A complementary pattern was revealed by BrdU labeling of pilidia, which shows dividing cells in the axils, near the stomach entrance, at the periphery of the apical organ, and a few other specific locations in the larval body (Figure 3). A 24-hour pulse of BrdU in a young feeding pilidium of *M. alaskensis* labeled about a dozen cells in each of the four pilidial axils (Figure 3A and B). We suggest that the BrdU-positive cells in the axils and their progeny are responsible for the majority of the growth in the pilidium larva, and produce the dark regions shown in Figures 1 and 2. In addition, several (approximately eight) BrdU-positive cells formed a ring at the rim of the apical organ (Figure 3A). We noted a corresponding dark region in the apical organ where the injected fluorescent label was diluted due to cell division (Figures 1E and 2C’). No BrdU-positive cells were found in the center of the apical organ (Figure 3), and, respectively, these cells remained brightly labeled with EMTB-3xGFP and FITC-dextran for many weeks, due to lack of cell division (Figure 1E). A pair of BrdU-positive cells was found at the entrance to the stomach (arrowhead 3 on Figure 3A); presumably, their progeny were responsible for the dark region near the stomach entrance in the EMTB-3xGFP and FITC-dextran-labeled larvae (Figure 1B, B”, C and 2C’). In this 24-hour pulse experiment BrdU also labeled a pair of mesenchymal cells inside the lappet ciliary band (arrowhead 1 on Figure 3A and B; arrowhead on Figure 3C), a pair of cells at the end of the buccal ridges (arrowhead 2 on Figure 3A and B) and two to three mesenchymal cells behind the buccal ridges (arrowhead 4 on Figure 3A). Collectively, these results demonstrate that the pilidium larva contains discrete proliferative zones which account for most of the growth of the larval body.

### Axillary stem cells contribute both to the larval body and the imaginal discs

The pilidium larva is a novel invention of the nemertean clade Pilidiophora [18-20] and its development, form and function is unique among animals [14,21]. The body of the nemertean worm develops inside the pilidium larva from a set of isolated rudiments, called imaginal discs, which grow inside the pilidium over the course of larval life and fuse around the stomach to form the juvenile body [14]. Most of the juvenile body is formed by two pairs of rudiments - the cephalic discs, which form the worm’s head, and the trunk discs, which form most of the rest of the worm (Figures 1B and 2D). These two main pairs of imaginal discs originate as invaginations of pilidial larval epidermis, notably, in the vicinity of the anterior axils (cephalic discs) and posterior axils (trunk discs); the cephalic discs are the first to form [14].

**Figure 4:**
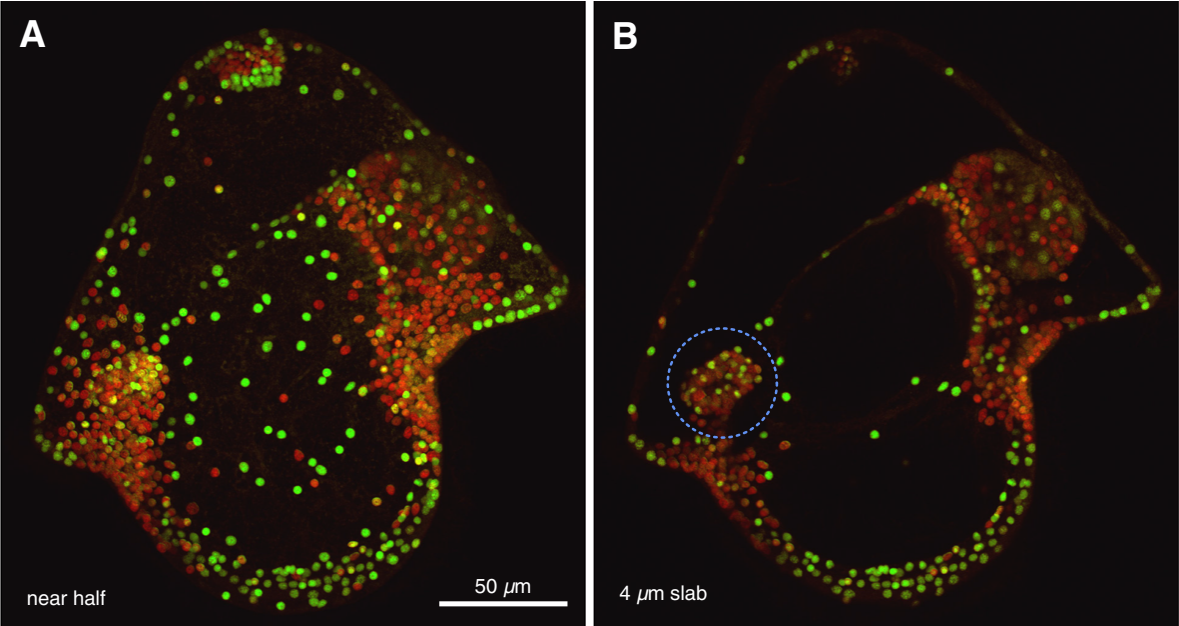
**Color-coding of relative BrdU retention, same dataset as Figure **3**D: 20-day-old pilidium labeled with six-hour BrdU treatment on day 6. (A)** The near half (projection of 45 0.8-μm sections); **(B)** a slab of five sections cutting through the cephalic imaginal disc (dashed outline). Confocal data was coded in Hue/Saturation/Brightness by calculating the simple ratio of Hoechst fluorescence to BrdU antibody fluorescence; the resulting images were used as the ‘Hue’ channel, running from red through green, whereas the original Hoechst image was used as the ‘Brightness’ channel. The ‘Saturation’ channel was full white. Consequently, all nuclei are shown, and redness indicates how much BrdU each nucleus retains. In other words, a green nucleus is one that either never incorporated any BrdU, or that diluted it to extinction through division; yellow nuclei clearly had an ancestor that was labeled during the BrdU pulse, but have since divided and diluted the label. The presence of many yellow and green nuclei in the cephalic disc, an organ which did not exist at the time of pulse labeling, shows not only that imaginal discs descend from axils, but that they include cells that divide enough to dilute BrdU to extinction.

To determine whether the pilidial axillary growth zones also contribute to the imaginal discs we conducted BrdU pulse-chase experiments. Young feeding pilidia of *M. alaskensis* (before the cephalic discs are detectable) were labeled with a six-hour pulse of BrdU, followed by a three-day chase, during which time the cephalic discs appeared. BrdU-positive cells were present inside the cephalic discs (Figure 3C), which suggests that cells from the anterior axils contribute to the cephalic discs because the only dividing cells in the vicinity are found in the anterior axils as shown by the BrdU-pulse experiment described above. Some of the axillary cells retain bright labeling after a pulse-chase (arrowhead in Figure 3C_2_), which suggests that they have not gone through many cell divisions after they were labeled; others, especially in the cephalic disc, retained substantially weaker labeling (arrowhead in Figure 3C_3_), which suggests that these cells went through several rounds of cell division. An extremely long (14-day) chase after a six-hour BrdU pulse in young feeding pilidia revealed a set of BrdU-positive cells (Figure 3D) in a pattern complementary to that seen in advanced pilidia labeled as zygotes with EMTB-3xGFP (Figure 1B) or FITC-dextran (Figure 2C and D): the BrdU-positive cells abound in the regions of the larval body that dilute the injected labels due to cell division. The cephalic discs themselves are composed primarily of BrdU-positive cells with the label diluted to various extents, and some cells that have no detectable BrdU in them (Figure 4). It is possible that these unlabeled cells simply escaped the initial BrdU pulse; however, given the spectrum of BrdU retention observed, it is more likely that they represent cells that have divided so many times that the label is no longer detectable.

### Axillary stem cells are small and uniciliated

**Figure 5:**
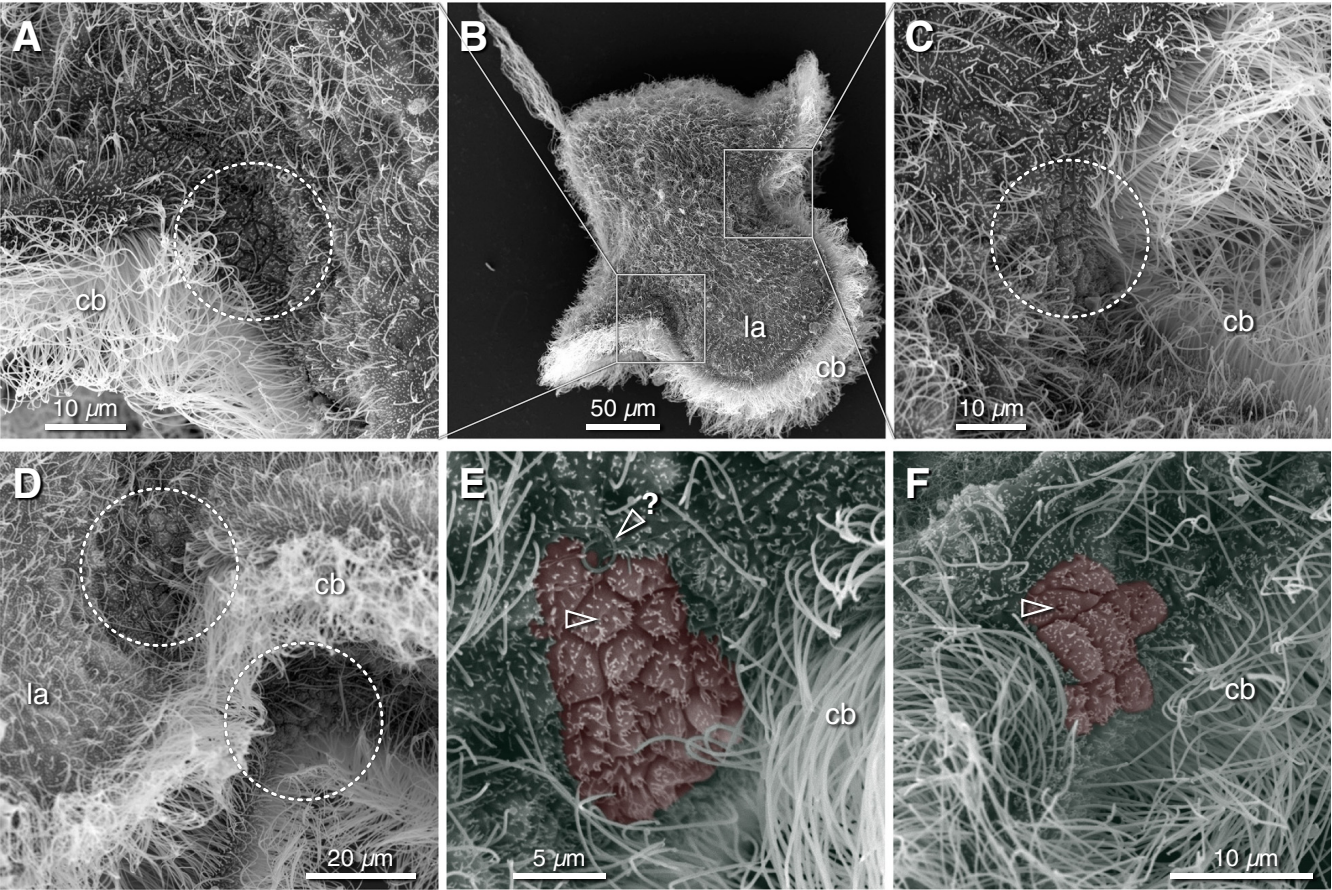
**Clusters of small uniciliate cells in the axils of the pilidium.** All: anterior left, apical up; la = lappet, cb = ciliary band. **(A-C)** Three-week-old pilidium of *M. alaskensis* (two to three pairs of discs stage): anterior axil **(A)**, overview **(B)**, and posterior axil **(C)**. Dashed circle indicates a cluster of approximately ten small, apparently non-ciliated cells. **(D)** Peering under the larval hat-brim, as it were, reveals that each outer axillary patch is matched by an equivalent inner patch; this instance shows the posterior axils, outer (left) and inner (right). **(E,F)** Higher-magnification views of outer posterior **(E)** and inner anterior **(F)** axils. Color highlighting shows those cells which lack motile cilia and appear to have each a single, very short ciliary rudiment (arrowheads). Highlighting may have missed some axillary cells in which the ciliary rudiment was absent or invisible. Question mark indicates a curled cilium borne by a cell with a small apex that also emits numerous long tendrils across the epithelium. One or a few of these curious cells are present near or within each outer axil.

BrdU labeling demonstrates that extensive, localized cell division enables the pilidium both to grow its larval body and develop the juvenile body inside. Because the predominant growth zones are the anterior and posterior axils, we looked for morphologically-distinct cells amongst the otherwise multiciliated epidermis. Scanning electron micrographs of two- to four-week-old pilidia of *M. alaskensis* revealed clusters of small cells (on the order of a dozen) in each of the pilidial axils (Figure 5). Each of these cells bears a single short, likely non-motile, cilium, as opposed to multiple long cilia on the surrounding epidermal cells. The position of these uniciliated cells corresponds to the position of the clusters of axillary cells that were BrdU-positive but with much-diluted label at a comparable developmental stage (Figures 3D and 4A). We suggest that these uniciliated cells are the stem cells, set aside during embryogenesis, that give rise to both the post-embryonic pilidial larval body and the juvenile body inside the pilidium via imaginal discs. Notably, there are two patches of stem cells per axil: one on the outer side, that is above or apical of the larval ciliated band, and one on the inner side, that is below, or vegetal of the larval ciliated band (Figure 5D). Cells of the inner and outer patches may have different fates. For example, one set may be contributing to the larval, and the other to the juvenile body. Alternatively, the outer cells may be forming the outer larval epidermis (the episphere), while the inner cells may be forming the inner larval epidermis (the hyposphere), lining the inside of the lappets and lobes and the buccal cavity. Based on the previous observation that the cephalic discs invaginate from the episphere and the trunk discs from the hyposphere [14], we hypothesize that the outer anterior axils and the inner posterior axils give rise, respectively, to those rudiments. Confirmation awaits development of methods for long-term lineage tracing in the pilidium.

## Conclusions

**Figure 6:**
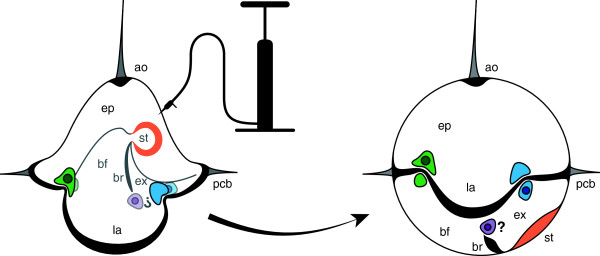
**Relative positions of axillary sites in the pilidium.** Left: diagrammatic pilidium in side view; apical organ (ao) and episphere (ep) up, lappets (la) down, primary ciliated band (pcb) defining larval margin. Inside are stomach (st) and buccal funnel (bf), divided from exhalant gutter (ex) by buccal ciliary ridges (br). Colored patches indicate axils, both outer (bold) and inner (faded), in the anterior (green), posterior (blue), and, hypothetically, at the end of the buccal ciliary ridges (purple). Darker circles within axils indicate those which give rise to imaginal discs. The fact that the hyposphere of the pilidium is almost entirely folded into the episphere at gastrulation obscures the relationship between axils; therefore, to the right we invite readers to imagine a spherical pilidium, that is, as if the blastocoel were inflated. Once the hyposphere is thus everted, such that the entire contiguous ectoderm and endoderm are laid out upon the surface of a sphere, the symmetry of the relation between anterior and posterior axils with respect to ciliated band and imaginal rudiments becomes plain. Again, the question mark indicates a hypothetical axil at the end of the buccal ridges; the cerebral organ discs originate at the end of the buccal ridges, and we detect both label dilution (Figure 1B, C) and BrdU incorporation (Figure 3A, B arrowhead 2, also Figure 3C) in these areas, but we have not spotted the expected population of uniciliated cells beneath the dense ciliation where the inner band of the lappets meets the end of the buccal ridges.

Our results clearly show that the nemertean pilidium larva, although primarily composed of multiciliated cells, grows and develops both the larval body and the rudiments of the juvenile inside via cell division in groups of small uniciliated stem cells located in specific regions of the larval body, which we refer to as the axils (summarized in Figure 6). The fact that stem cells are restricted to specific growth zones likely has consequences for larval regeneration. For example, these results would predict that the pilidium larva should be able to regenerate its lobes and lappets and the associated primary ciliated band (as long as the axils remain intact), but unable to regenerate the apical organ (as long as the entire organ is removed).

Furthermore, our results show that the juvenile body inside the pilidium is formed largely by the progeny of the same group of cells that allow the pilidium to grow. Some years ago, in a provocative review, Davidson *et al*. [22] suggested that the body plans of modern-day animal larvae might be more or less like the original metazoans; that larval bodies are essentially eutelic and that the key innovation resulting in the explosive diversification of animal body plans - today’s adults - during the Cambrian was the invention of ‘set-aside cells’ that remain proliferative and pluripotent (stem cells, that is). Although our one-sentence summary unjustly oversimplifies this scheme, the ‘set-aside cell’ hypothesis has not accrued much support from closer examination of extant life histories. The pilidium, for example, epitomizes ‘maximally-indirect development’, but recent work shows that this larval form is a novel body intercalated into the life history [18,19]. And although we affirm that most of the pilidium’s larval body as produced by embryogenesis is indeed proliferatively-limited, the very ‘set-aside cells’ that create the imaginal discs also add extensively to the larval body. Yet the central insight prompting the ‘set-aside cell’ hypothesis remains intriguing: that the evolution of development is largely to do with adapting strategies for balancing proliferation with differentiation while optimizing function. Nowhere is this so stark as in the apparent dichotomy between ciliogenesis and mitosis. This factor profoundly shapes the landscape of cell differentiation in animal embryos and larvae, and likely in mature tissues as well. Why animal cells should be so constrained is not immediately clear. The obvious answer - that the constraint is due to the shared duty of the centriole as both basal body and spindle pole - explains little, because many protists divide while ciliated, some even while multiciliated, despite the same dual role for the centriole. Curiously, most other multicellular eukaryotes either lack cilia (for example, fungi and rhodophytes) or abandon ciliation during the multicellular phase (brown algae; land plants); besides animals, the other exception are the Volvocales which have their own mechanistically-distinct constraint that keeps mitosis and ciliation separate [23]. Does the freedom to swim away undermine cooperation?

## Abbreviations

EMTB: Ensconsin microtubule binding domain; GFP: green fluorescent protein; FSW: filtered seawater; FITC: fluorescein isothiocyanate; BrdU: bromo-deoxyuridine.

## Competing interests

The authors declare that they have no competing interests or conflicts.

## Authors’ contributions

AMB: data collection and analysis, critical revision and final approval of the manuscript. GvD: conception and design, data collection and analysis, figure preparation, critical revision and final approval of the manuscript. SAM: conception and design, data collection and analysis, manuscript writing, financial support, and final approval of the manuscript. All authors read and approved the final manuscript.
